# Gait Stability Has Phase-Dependent Dual-Task Costs in Parkinson’s Disease

**DOI:** 10.3389/fneur.2018.00373

**Published:** 2018-05-30

**Authors:** Peter C. Fino, Martina Mancini, Carolin Curtze, John G. Nutt, Fay B. Horak

**Affiliations:** ^1^Department of Neurology, Oregon Health & Science University, Portland, OR, United States; ^2^Veterans Affairs Portland Health Care System, Portland, OR, United States

**Keywords:** lyapunov exponents, locomotion, cognitive dual-task, local dynamic stability, dynamic postural control

## Abstract

Dual-task (DT) paradigms have been used in gait research to assess the automaticity of locomotion, particularly in people with Parkinson’s disease (PD). In people with PD, reliance on cortical control during walking leads to greater interference between cognitive and locomotor tasks. Yet, recent studies have suggested that even healthy gait requires cognitive control, and that these cognitive contributions occur at specific phases of the gait cycle. Here, we examined whether changes in gait stability, elicited by simultaneous cognitive DTs, were specific to certain phases of the gait cycle in people with PD. Phase-dependent local dynamic stability (LDS) was calculated for 95 subjects with PD and 50 healthy control subjects during both single task and DT gait at phases corresponding to (1) heel contact—weight transfer, (2) toe-off—early swing, and (3) single-support—mid swing. PD-related DT interference was evident only for the duration of late swing and LDS during the heel contact—weight transfer phase of gait. No PD-related DT costs were found in other traditional spatiotemporal gait parameters. These results suggest that PD-related DT interference occurs only during times where cortical activity is needed for planning and postural adjustments. These results challenge our understanding of DT costs while walking, particularly in people with PD, and encourage researchers to re-evaluate traditional concepts of DT interference.

## Introduction

Locomotor deficits have been widely reported in people with Parkinson’s disease (PD) due to the degeneration of basal ganglia and brainstem structures that contribute to control of gait and balance (1–5). To compensate for disrupted subcortical pathways, individuals with PD exhibit more goal-directed locomotion (6–8), with greater reliance on cortical networks when walking (8, 9). In particular, people with PD exhibit increased gait variability (10–13) and abnormal gait dynamics (i.e., how gait parameters vary over time) (14), often attributed to this loss of automaticity and increased cortical control of locomotion (7, 8, 15).

The primary evidence for this compensatory cognitive control in people with PD stems from excessive dual-task (DT) cost (16, 17). DT paradigms involve a cognitive task performed concurrently with a locomotor task, producing interference between the tasks and leading to decreases in the performance of one or both tasks (18–20). While there are several prevailing theories to describe the nature of these performance deficits, called DT costs or dual-task changes (DTC) (21, 22), a common notion maintains that the regulation of the cognitive task and the regulation of gait interfere with one another. In healthy people, walking normally requires little cortical attention and therefore shows little DTCs, whereas in people with PD, walking requires significant cortical compensation, resulting in large DTCs (18–20, 23, 24).

Larger DTCs in spatiotemporal measures such as stride time, stride length, and gait speed have been reported in people with PD compared to control subjects (25), and larger DTCs have been associated with PD severity (19, 26), or freezing of gait (27, 28). However, these spatiotemporal measures of gait do not separate specific phases within the gait cycle that may be critical to stable locomotion. For instance, electroencephalography studies have indicated that gait involves cortical contributions at specific phases to plan the next foot placement, transfer weight from one step to the next, and maintain stability (29–31). Therefore, the interference between the cognitive task demands and the compensatory cortical control of gait may be specific to certain phases of the gait cycle that depend most on cortical control.

To investigate whether people with PD have DTCs that are specific to certain phases of the gait cycle, we examined phase-dependent measures of gait stability and traditional spatiotemporal gait measures in subjects with idiopathic PD and healthy age-matched control subjects during self-paced, over-ground walking with and without a cognitive DT. Phase-dependent local dynamic stability (LDS) of trunk movements was calculated at three phases of the gait cycle, corresponding to: (1) heel contact—weight transfer, (2) toe-off—early swing, and (3) single-support—mid swing. Phase-dependent LDS quantifies the rate at which local perturbations are attenuated during specific phases of the gait cycle (32). Previous studies have shown that phase-dependent LDS during weight transfer, but not other phases, is a sensitive predictor of falls in elderly populations and can differentiate gait in young and older adults, suggesting that dynamic stability during weight transfer, specifically, is sensitive to neuromotor changes related to fall risk due to aging (32–35).

Greater knowledge of how cognitive tasks interfere with locomotor demands in people with PD may facilitate targeted intervention strategies that focus on specific, highly affected components of gait. Therefore, the purpose of this study was to examine if people with PD exhibit phase-specific DTCs in local dynamic gait stability. We hypothesized that people with PD would exhibit the most severe DTC in dynamic stability during the weight transfer phase of gait compared with controls. We anticipated that these PD-related DTCs in dynamic stability associated with weight transfer would differ between subjects with PD and healthy control subjects while the DTCs in other spatiotemporal gait would not.

## Materials and Methods

### Participants

As part of a larger study (Clinical Trials NCT02231073 and NCT02236286), 100 individuals with idiopathic PD were recruited for this baseline analysis. All subjects with PD had clinically diagnosed idiopathic PD by a neurologist and were tested in the practical OFF levodopa state, after withholding anti-parkinsonian medication for ≥12 h. Inclusion criteria for subjects with PD were (1) between 50 and 90 years old, (2) no major musculoskeletal or peripheral disorders (other than PD) that could significantly affect their balance and gait, (3) ability to stand and walk unassisted, and (4) met criteria for idiopathic PD according to the according to the Brain Bank Criteria for PD (36). In addition, 56 healthy elderly adults were recruited from the community. Exclusion criteria for both groups were as follows: any other neurological disorders or musculoskeletal impairments that interfere with gait or balance, and inability to follow instructions.

Five individuals with PD and six healthy controls were excluded from the final analysis due to technical considerations (see Analysis). Demographic characteristics for subjects retained in the final analysis for each group are provided in Table 1. This study was carried out in accordance with the recommendations of the Oregon Health & Science University (OHSU) and Veterans Affairs Portland Health Care System (VAPORHCS) joint institutional review board (IRB) with written informed consent from all subjects. All subjects gave written informed consent in accordance with the Declaration of Helsinki. The protocol was approved by the OHSU (#4131) and the OHSU/VAPORHCS joint IRB (#8979).

**Table 1:** Demographic data.

	Controls	PD	*p*-Value
N	50	95	
% Female	38	32	
Age (years)	67.8 (8.0)	68.7 (7.7)	0.947
Height (cm)	171.7 (9.8)	174.2 (10.2)	0.176
Mass (kg)	73.8 (14.6)	79.5 (15.2)	**0.033**
miniBEST	24.6 (2.2)	18.4 (4.7)	**<0.001**
TUG time (s)	18.3 (3.1)	23.2 (10.0)	**0.001**
MoCA	26.8 (1.9)	25.5 (3.6)	**0.019**
SCOPA-COG	32.0 (3.5)	28.1 (5.6)	**<0.001**
Fall in the past year (%)	12	38	
Disease duration (years)	–	7.0 (5.2)	
MDS-UPDRS part III	–	40.4 (12.9)	
PIGD score	–	5.0 (3.2)	
H&Y (range)	–	2–3	
N with freezing of gait	–	26	

*Where applicable, groups were compared using independent sample t-tests and a significance level of 0.05*.

*Bold values indicate significant differences between PD and control subjects*.

*PD, Parkinson’s disease; miniBEST, mini Balance Evaluation Systems Test; MoCA, Montreal Cognitive Assessment; PIGD, Posture Instability and Gait Disability*.

### Procedures

Subjects with PD were clinically rated by a trained examiner on the Motor Section (III) of the Unified PD Rating Scale (MDS-UPDRS), which consists of 23 items related to bradykinesia, rigidity, tremor, and posture and gait signs rated on a four-point scale (37), prior to the mobility assessment. The Posture Instability and Gait Disability (PIGD) subscore was also calculated from the MDS-UPDRS Part III (38).

At the beginning of the mobility assessment, each participant performed a seated cognitive task of reciting every other letter of the alphabet for 1 min. The number of total responses and the number of correct responses were recorded. Each participant was then outfitted with eight inertial sensors (APDM, Inc., Portland, OR, USA), worn on the sternum, lumbar spine, bilaterally on the wrists, anterior distal region of the shanks, and feet. Each inertial sensor recorded tri-axial accelerations and angular velocities at 128 Hz. Data from the wrist-sensors were not used for this study. As part of the larger study, participants completed several tests of balance and mobility, including the Timed Up and Go (TUG), mini Balance Evaluation Systems Test (miniBEST), and self-paced walking trials (Table 1). In addition, each participant completed the Montreal Cognitive Assessment (MoCA) (39) and SCOPA-COG.

Analysis of phase-dependent gait stability was based on two self-paced, walking trials: one 2-min trial with no added cognitive task [single-task (ST)] and one 1-min trial with a simultaneous cognitive task (DT). In both conditions, participants were instructed to walk at a comfortable pace back and forth continuously between two lines 7.62 m apart. In the ST condition, participants were instructed to walk for the entire 2 min; no other task was given. In the DT condition, participants were instructed to walk for 1 min while reciting every other letter of the alphabet. The order of the conditions was not randomized; the ST condition was always completed before the DT condition. In the DT condition, participants were given no instruction regarding the prioritization of one task over the other. The number of correct responses during the DT condition was recorded.

### Analysis

Raw 3-D accelerometer and gyroscope data were extracted from the sternum, lumbar spine, and shank inertial sensors for each walking trial. Each walking trial was segmented into multiple, straight walking bouts by removing turns and removing one stride immediately preceding and following each turn. Turns were identified using a threshold-based detection algorithm based on the axial angular velocity of the lumbar sensor (40). Heel-contact, toe-off, and mid-swing events were detected using the angular velocity of the shank as described by Salarian et al. (41). Each straight walking bout was then divided into non-overlapping segments of five consecutive, straight walking strides, with each stride time-normalized to 130 points to maintain equal data-length across segments. If a walking bout did not include at least five straight strides, it was excluded from the remainder of the analysis. Subjects were excluded entirely if they had no walking bouts with at least five consecutive straight strides in either the ST or DT conditions.

Phase-dependent LDS was calculated for each walking bout of five strides at three phases within the gait cycle, heel contact—weight transfer; toe off—early swing; and single-support—mid swing, based on procedures described by Ihlen et al. (34). Briefly, a 6D state space ***X***(*t*) = [*a*_AP_(*t*), *a*_ML_(*t*), *a*_ML_(*t*), ω_AP_(*t*), ω_ML_(*t*), ω_VT_(*t*)] was constructed using the 3D trunk accelerations *a*(*t*) and 3D trunk angular velocities ω(*t*) from the sternum inertial sensor. Next, points corresponding to heel-contact, toe-off, and mid-swing events were found within the state space, and two nearest neighbors within the space were identified for each event. For each gait event, the average distances from the trajectories of the two nearest neighbors to the trajectory of gait event were tracked for one step, and mean log divergence curves were created by mapping the average distance across all similar gait events (e.g., all heel-contact events, all toe-off events, and all mid-swing events) as a function of the percentage of normalized stride. Phase-dependent LDS was then estimated for each segment using maximum finite-time Lyapunov exponents calculated from the slope of the mean log divergence curves from the initial gait event to the next 10% of the step cycle (i.e., 5% of the gait cycle) for each phase, heel contact (λ_HC_), toe off (λ_TO_), and mid swing (λ_MS_) (e.g., heel contact + 5% of gait cycle, toe off + 5% of gait cycle, and mid swing + 5% of the gait cycle, respectively). This procedure can be described mathematically using the following equation:

$$\lambda_{\text{bout}}=\frac{{\langle \text{ln }\langle d_{i}(t)\rangle \rangle }_{\text{step}}}{t},$$

where ⟨*d_i_*(*t*)⟩ is the average Euclidean distance between the *i* nearest neighbor trajectories and the reference trajectory at each point in time *t*, where the gait event (e.g., heel contact, toe off, or mid swing) defined *t* = 0 within the state space, ⟨…⟩_step_ is the average over all steps within the bout, and λ_bout_ is the estimate of phase-dependent LDS for a single bout. The median λ_HC_, λ_TO_, and λ_MS_ across all walking bouts was used as the final estimate of phase-dependent LDS at heel contact, toe off, and mid swing, respectively.

Traditional LDS, λ_Kantz_, was also calculated for each walking segment of five time-normalized strides following Kantz’s algorithm (42) and previous reports for estimating local dynamics stability over short bouts of gait (43–45). A 9D state space was constructed from the three-dimensional trunk accelerations and their twice time-delayed copies using a fixed time delay of 0.25 of the average stride time. For each point, the average distance to the two nearest neighbors of the trajectory were tracked for one step, and mean log divergence curves were created by mapping the average distance across all points as a function of the percentage of normalized stride. Traditional LDS, λ_Kantz_, was then estimated for each segment using maximum finite-time Lyapunov exponents calculated from the slope of the mean log divergence curves from the 0 to 0.5 strides, and the median across all walking segments was used as the final estimate of λ_Kantz_. For all four stability outcomes, greater values of λ indicate faster divergence or nearby trajectories in state space and are therefore associated with less stability; smaller values of λ indicate slower divergence and are typically associated with increased stability (46, 47).

To compare the DTC of stability outcomes to the DTC of traditional gait measures, temporal gait measures of stride time, double support time, early swing time (toe off to mid swing), and late swing time (mid swing to heel contact) were calculated from the difference in time between respective gait events. Gait speed and stride length were calculated from Mobility Lab software using analysis version 3.0 (Mobility Lab v2, APDM, Inc., Portland, OR, USA).

To evaluate the performance on the cognitive task, the total number of responses and the number of correct responses were tabulated for both the seated and DT walking conditions. Accuracy was calculated as the number of correct responses divided by the total number of responses $\left(\text{Accuracy }=\frac{\#\text{Correct}}{\text{Total}}\right)$. For cognitive task outcomes of total responses, correct responses, and accuracy, the DTC was calculated as the change relative to seated.

### Statistical Analysis

Independent sample *t*-tests compared age, height, mass, mini-BEST scores, MoCA scores, and SCOPA-COG scores between the PD and control groups. To investigate whether outcomes differed between groups, linear mixed models were fit for each stability outcome (λ_HC_, λ_TO_, λ_MS_, and λ_Kantz_), spatiotemporal measure of gait (gait speed, stride length, stride time, double support time, early swing time, and late swing time), and cognitive task outcome (total responses, correct responses, and accuracy). Each model was adjusted for group, task (ST versus DT), and the group*task interaction. The group*task interaction term was included in each model to test whether groups had different linear DTC between task conditions. Each model included a random intercept for each subject to account for the repeated measurements within each subject. For the cognitive outcomes, the task effect compared seated to walking conditions. Gait speed was included as a covariate in models for stability outcomes to account for variations in stability with gait speed (48, 49).

To confirm that any significant group*task interaction was robust to methods of calculating DTC (23), we performed *post hoc* analyses on any outcome with a significant group*task interaction. As the group*task interaction term in the linear mixed models assessed the linear DTC between tasks (DT − ST), group differences in the DTC as a percentage $\left(\%\text{DTC }=\;\frac{\text{DT}-\text{ST}}{\text{ST}}\times 100\%\right)$ were tested using independent sample *t*-tests. To limit the number of comparisons, the comparison of %DTC between groups was only performed on outcome measures with a significant group*task interaction.

To assess whether DTCs were associated with disease duration, severity, or cognitive function in PD, Spearman correlation coefficients compared the %DTCs of each outcome with a significant group*task interaction to disease duration, the MDS-UPDRS Part III subscore, the PIGD score from the MDS-UPDRS, the miniBEST score, the MoCA score, and the SCOPA-COG score. All statistical analysis was performed in MATLAB r2017a (The Mathworks Inc., Natick, MA, USA) using the Statistics and Machine Learning Toolbox. A significance level of 0.05 was used throughout.

## Results

Ninety-five subjects with PD and 50 healthy control subjects were retained in the final analysis after excluding five subjects with PD and six control subjects with no bouts of at least five strides during both ST and DT gait. The PD and control groups had medians (IQR) of 12 (2) and 14 (3) bouts of ST gait, respectively, and 6 (1) and 6 (2) bouts of DT gait, respectively, included in the analysis. There were no significant differences between groups in age or height. The PD group had significantly greater mass, lower miniBEST, MoCA, and SCOPA-COG scores, and had slower TUG times (Table 1). Univariate descriptive statistics for each outcome are shown in Table 2.

**Table 2:** Univariate means (SD) of each outcome stratified by group and condition.

	Single task		Dual task	
	Mean	SD	Mean	SD
**Gait speed (m/s)**				
Control	1.12	0.14	0.95	0.17
PD	0.93	0.20	0.78	0.19
**Stride length (m)**				
Control	1.19	0.08	1.11	0.10
PD	0.99	0.19	0.89	0.19
**Stride time (s)**				
Control	1.04	0.13	1.07	0.15
PD	1.07	0.16	1.09	0.15
**Time in double support (%)**				
Control	22.5	3.9	24.6	3.9
PD	23.9	4.8	26.7	6.2
**Time in early swing (%)**				
Control	51.2	4.8	48.1	5.4
PD	49.2	5.6	46.4	6.4
**Time in late swing (%)**				
Control	26.5	3.3	27.4	3.3
PD	27.0	3.3	27.0	3.7
**Total cognitive responses (n)^a^**				
Control	36	7	35	8
PD	32	10	29	8
**Correct cognitive responses (n)^a^**				
Control	34	8	31	8
PD	29	11	26	9
**Cognitive task accuracy (%)^a^**				
Control	93	9	89	10
PD	90	11	88	11
**λ_HC_**				
Control	0.15	0.04	0.13	0.04
PD	0.13	0.04	0.12	0.04
**λ_TO_**				
Control	0.07	0.02	0.08	0.02
PD	0.08	0.02	0.08	0.02
**λ_MS_**				
Control	0.11	0.02	0.11	0.02
PD	0.12	0.02	0.11	0.02
**λ_Kantz_**				
Control	0.30	0.07	0.34	0.08
PD	0.35	0.07	0.37	0.09

*^a^ST condition for cognitive responses refers to the seated condition*.

*PD, Parkinson’s disease*.

**Figure 1:**
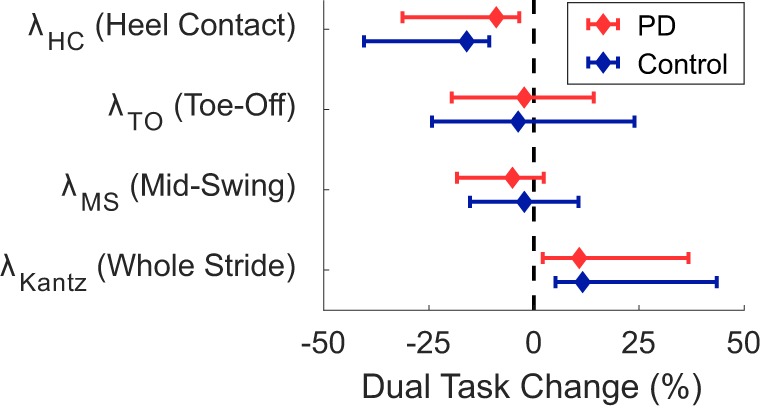
Median dual-task changes (DTC) as percentages (%DTC) and IQRs for phase-dependent local dynamic stability (LDS) measures calculated at (1) heel contact—weight transfer, λ_HC_, (2) toe off—early swing, λ_TO_, and (3) single-support—mid swing, λ_MS_, and traditional LDS calculated at all points within a stride, λ_Kantz_. DTCs were calculated as a percentage change with respect to single-task gait. *Phase-dependent LDS during heel contact—weight transfer, λ_HC_, was significantly different between groups whether calculated as a linear change (see Table 3), or as a percentage change. Group differences in DTCs as percentages were not tested on other stability outcomes as the group*task interactions were not significant in the initial linear mixed models.

A significant group*task effect was found for phase-dependent stability at weight transfer (λ_HC_) meaning that subjects with PD became less dynamically stable in the DT condition relative to the difference between conditions in the control subjects (Table 3, Figure 1). No group*task effect was found for stability at other phases (λ_TO_, λ_MS_) or when assessed without regards to phase (λ_Kantz_). Phase-dependent stability at weight transfer (λ_HC_) and mid swing (λ_MS_) was significantly greater (i.e., less stable) with faster gait speeds, while non-phase-dependent stability (λ_Kantz_) was significantly lower (i.e., more stable) with faster gait.

**Table 3:** Results from the linear mixed models for each stability measure.

	Beta	SE	Lower CI	Upper CI	*p*-Value
**λ_HC_**					
Intercept	0.072	0.015	0.042	0.103	<0.001
**Task (ref ST)**	**−0.011**	**0.005**	**−0.020**	**−0.001**	**0.024**
**Gait speed**	**0.070**	**0.013**	**0.045**	**0.096**	**<0.001**
Group (ref controls)	−0.006	0.007	−0.019	0.007	0.390
Group*Task	**0.011**	**0.005**	**0.000**	**0.021**	**0.043**
**λ_TO_**					
Intercept	0.077	0.009	0.060	0.094	<0.001
Task (ref ST)	0.001	0.003	−0.006	0.008	0.756
Gait speed	−0.003	0.007	−0.017	0.011	0.681
**Group (ref controls)**	**0.008**	**0.004**	**0.001**	**0.012**	**0.029**
Group*Task	−0.003	0.004	−0.011	0.004	0.412
**λ_MS_**					
Intercept	0.072	0.009	0.054	0.089	<0.001
Task (ref ST)	0.004	0.003	−0.002	0.010	0.163
**Gait speed**	**0.031**	**0.008**	**0.016**	**0.046**	**<0.001**
**Group (ref controls)**	**0.015**	**0.004**	**0.008**	**0.023**	**<0.001**
Group*Task	−0.005	0.003	−0.011	0.002	0.178
**λ_Kantz_**					
Intercept	0.471	0.031	0.410	0.533	0.000
Task (ref ST)	0.009	0.013	−0.017	0.035	0.486
**Gait speed**	**−0.151**	**0.026**	**−0.203**	**−0.100**	**<0.001**
Group (ref controls)	0.020	0.014	−0.007	0.048	0.142
Group*Task	−0.011	0.015	−0.041	0.018	0.452

*Lower and upper 95% confidence intervals (CI) for beta are also presented. Bold values indicate significant effects at *p* < 0.05*.

**Figure 2:**
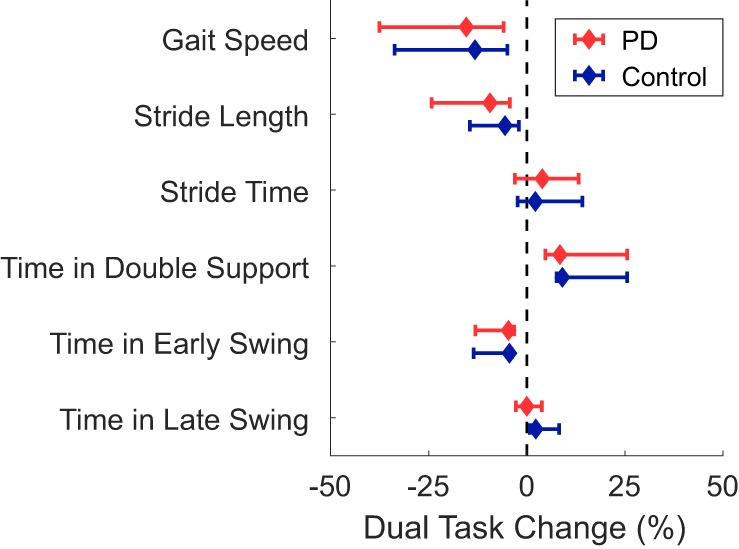
Median dual-task changes (DTC) as percentages (%DTC) and IQRs for spatiotemporal measures of gait for people with Parkinson’s disease (PD) (red) and healthy control subjects (blue). DTCs were calculated as a percentage change with respect to single-task gait. *Time spent in late swing was significantly different between groups whether calculated as a linear change (see Table 4), or as a percentage change. Group differences in DTCs as percentages were not tested on other spatiotemporal outcomes as the group*task interactions were not significant in the initial linear mixed models.

A significant group*task interaction effect was found for time spent in the late swing phase, where, relative to the ST condition, control subjects increased the time spent in late swing in the DT condition but subjects with PD did not change. No other spatiotemporal measure had a significant group*task interaction effect indicative of PD-related DTCs. Subjects with PD had significantly slower gait speed, shorter stride lengths, and spent less time in early swing compared to controls (Table 4; Figure 2). The DT condition led to slower gait speeds, shorter stride lengths, more time spent in double support, and less time in early swing compared to the ST condition across all subjects.

**Table 4:** Results from the linear mixed models for each spatiotemporal measure.

	Beta	SE	Lower CI	Upper CI	*p*-Value
**Gait speed (m/s)**					
Intercept	1.12	0.03	1.07	1.17	<0.001
**Task (ref ST)**	**−0.17**	**0.02**	**−0.20**	**−0.14**	**<0.001**
**Group (ref controls)**	**−0.20**	**0.03**	**−0.26**	**−0.13**	**<0.001**
Group*Task	0.02	0.02	−0.02	0.06	0.368
**Stride length (m)**					
Intercept	1.19	0.02	1.15	1.24	<0.001
**Task (ref ST)**	**−0.08**	**0.01**	**−0.11**	**−0.06**	**<0.001**
**Group (ref controls)**	**−0.20**	**0.03**	**−0.26**	**−0.14**	**<0.001**
Group*Task	−0.02	0.01	−0.05	0.00	0.090
**Stride time (s)**					
Intercept	1.04	0.02	0.99	1.08	<0.001
Task (ref ST)	0.03	0.03	−0.02	0.08	0.230
Group (ref controls)	0.04	0.03	−0.02	0.09	0.157
Group*Task	−0.02	0.03	−0.08	0.04	0.532
**Time in double support (%)**					
Intercept	22.5	0.7	21.1	23.9	<0.001
**Task (ref ST)**	**2.1**	**0.4**	**1.3**	**3.0**	**<0.001**
Group (ref controls)	1.4	0.9	−0.3	3.1	0.107
Group*Task	0.6	0.5	−0.4	1.7	0.231
**Time in early swing (%)**					
Intercept	51.1	0.8	49.6	52.7	<0.001
**Task (ref ST)**	**−3.0**	**0.5**	**−4.0**	**−2.0**	**<0.001**
**Group (ref controls)**	**−2.0**	**1.0**	**−3.9**	**−0.0**	**0.048**
Group*Task	−0.2	0.6	−0.9	1.4	0.732
**Time in late swing (%)**					
Intercept	26.5	0.5	25.6	27.5	<0.001
**Task (ref ST)**	**0.9**	**0.3**	**0.3**	**1.4**	**0.002**
Group (ref controls)	−0.5	0.6	−0.7	1.6	0.431
Group*Task	**−0.9**	**0.3**	**−1.5**	**−0.2**	**0.010**

*Lower and upper 95% confidence intervals (CI) for beta are also presented. Bold values indicate significant effects at *p* < 0.05*.

**Figure 3:**
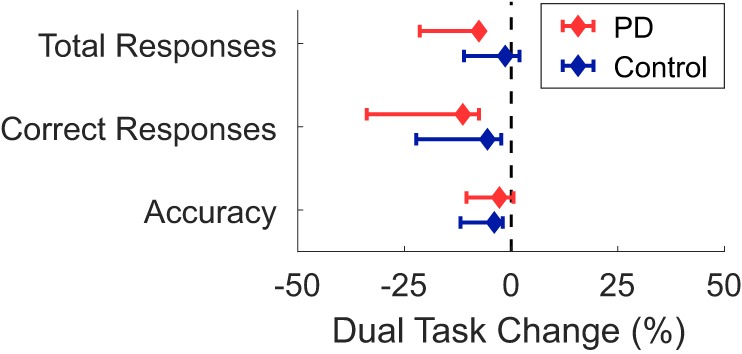
Median dual-task changes (DTC) as percentages (%DTC) and IQRs for cognitive outcomes for people with Parkinson’s disease (PD) (red) and healthy control subjects (blue). DTCs were calculated as a percentage change with respect to seated. Group differences in DTCs as percentages were not tested for any cognitive outcome as no group*task interactions were significant in the initial linear mixed models.

There was no significant group*task interaction for any cognitive task outcome (Table 5; Figure 3). Main effects of group and task were found for the number of correct responses, but not for the number of total responses or accuracy. Subjects with PD had fewer correct responses than the control group across both conditions, and the walking condition had fewer correct responses than the seated condition across both groups.

**Table 5:** Results from the linear mixed models for measures of cognitive task performance.

	Beta	SE	Lower CI	Upper CI	*p*-Value
**Number of total responses**					
Intercept	35.99	1.23	33.45	38.30	<0.001
Task (ref ST)	−1.30	0.79	−2.86	0.26	0.103
**Group (ref controls)**	**−3.86**	**1.53**	**−6.87**	**−0.86**	**0.012**
Group*Task	−1.07	1.00	−3.00	0.92	0.299
**Number of correct responses**					
Intercept	33.5	1.29	30.96	36.04	<0.001
**Task (ref seated)**	**−2.60**	**0.81**	**−4.19**	**−1.02**	**0.001**
**Group (ref controls)**	**−4.29**	**1.60**	**−7.45**	**−1.14**	**0.008**
Group*Task	−0.29	1.01	−2.28	1.69	0.771
**Cognitive task accuracy (%)**					
Intercept	92.5	14.7	89.6	95.4	<0.001
**Task (ref seated)**	**−3.4**	**1.3**	**−5.9**	**−0.8**	**0.010**
Group (ref controls)	−2.6	1.8	−6.2	0.9	0.149
Group*Task	−1.4	1.6	−1.8	4.6	0.399

*Lower and upper 95% confidence intervals (CI) for beta are also presented. Bold values indicate significant effects at *p* < 0.05*.

*Post hoc t*-tests were only performed on the %DTC for two outcomes, λ_HC_ and time spent in late swing, as those were the only outcomes with significant group*task interactions in the linear mixed models. The %DTC for λ_HC_ was significantly smaller in subjects with PD compared with controls (*t* = −2.56, *p* = 0.012). Similarly, the %DTC for time spent in late swing was significantly smaller in subjects with PD compared to controls (*t* = −2.78, *p* = 0.006).

**Figure 4:**
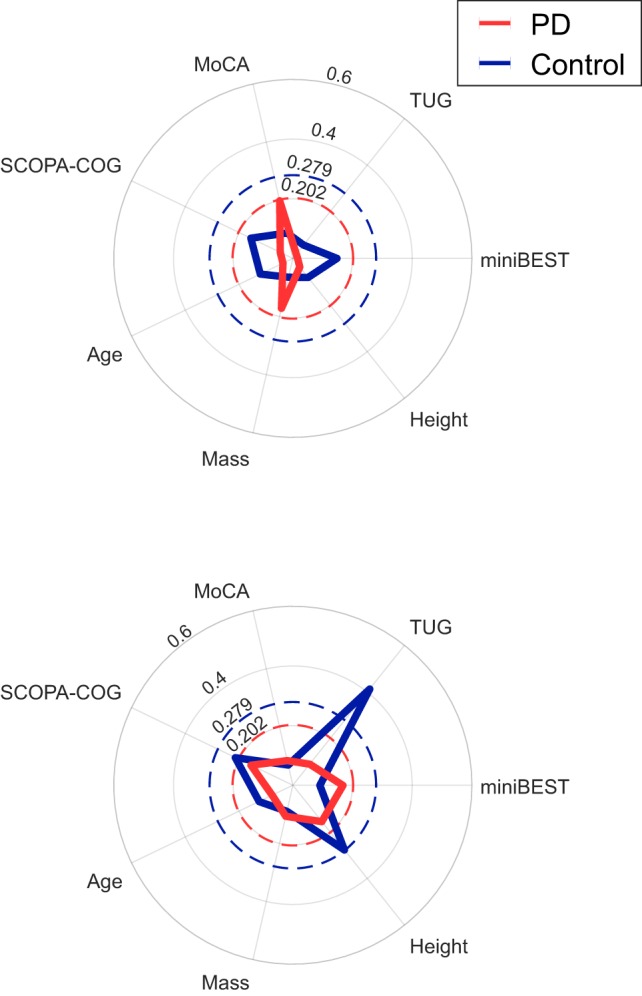
Radar plots of the absolute value of Spearman’s correlation coefficients between demographic and clinical outcomes and the %DTCs of λ_HC_ (top) or time in late swing (bottom). The dashed red and blue circles indicate the critical ρ value for *p* = 0.05 for Parkinson’s disease (PD) and control groups, respectively. The %DTC for time in late swing was significantly associated with TUG time in controls only (ρ = 0.41, *p* = 0.004). There were no other significant correlations (*p* > 0.05).

The %DTC for time in late swing was significantly associated with TUG time in controls only (ρ = 0.41, *p* = 0.004), but not in subjects with PD. The %DTCs of λ_HC_ and time in late swing were not significantly associated with disease duration, MDS-UPDRS Part III subscore, or UPDRS PIGD score in subjects with PD (Table 6). The %DTCs of λ_HC_ and time in late swing were not associated with miniBEST, MoCA, or SCOPA-COG scores, or with age, height, or mass in either group (Figure 4).

**Table 6:** Spearman correlation coefficients and *p*-values for comparisons between the %DTC of λ_HC_ and clinical characteristics in subjects with Parkinson’s disease.

	Disease duration		MDS-UPDRS part III		Posture Instability and Gait Disability	
			
	ρ	*p*-Value	ρ	*p*-Value	ρ	*p*-Value
%DTC λ_HC_	−0.011	0.917	0.091	0.384	0.063	0.549
%DTC time in late swing	0.039	0.709	0.075	0.475	0.109	0.294

## Discussion

We compared the DTC on phase-dependent LDS during phases of the gait cycle beginning with heel contact, toe off, and mid swing in people with PD and healthy matched controls. Compared to controls, people with PD only demonstrated greater DTCs during the phase beginning at heel contact and corresponding to the weight transfer phase of gait. Many previous studies have described larger DTCs on spatiotemporal measures of gait in people with PD compared to controls [see review by Kelly et al. (25)], but these measures cannot examine intra-stride patterns. Our results suggest that cognitive DTs affect gait stability in an intra-stride, phase-specific pattern in people with PD.

Increasing evidence has suggested that gait has intermittent contributions from the cortex, and that these contributions are locked to specific phases of the gait cycle (29, 31, 50–52). Cortical activity in the premotor cortex is highest during single-limb stance prior to heel contact, representative of cortical planning of balance control and foot placement (30, 50). Others have reported elevated activity in the sensorimotor cortices during weight transfer (31, 52, 53), indicative of sensory feedback for state estimation of postural adjustments (54). While it is not clear how variations in cortical activity affect DT interference, our results suggest DTC can similarly fluctuate within a gait cycle.

We found significant DTCs, indicated by a significant main effect of task, in both the PD and control groups with slower gait speeds, shorter stride lengths, more time spent in double support, and less time spent in early swing compared to the ST condition. However, these DTCs did not differ between subjects with PD and healthy control subjects. Previous work by Rochester et al. (23) suggested that DT deficits in PD stem from two underlying causes: age-related DT declines in overall gait performance and PD-related DT deficits in specific measures of postural control. Specifically, PD-related DT deficits were apparent only in step width and step width variability (23), implying that cognitive tasks only have PD-related interference with measures pertaining to the unstable mediolateral (ML) direction during gait (55, 56). Stable gait is largely achieved by placing the swing limb to redirect the lateral movement of the center-of-mass (57, 58). While weight transfer occurs after placement of the swing limb, planning the placement of the swing limb occurs during second half of the swing phase (59), during a period of elevated activity in the premotor cortex (50). In a study of healthy elderly, Bruijn et al. (30) found that stabilizing healthy young participants in the ML direction significantly decreased step width, improved trunk stability, and reduced the activity in the premotor cortex immediately before and during weight transfer. Therefore, it appears that stability, particularly in the ML direction, might require significant activity from the premotor and supplementary motor areas (SMA) for correct limb placement and weight transfer. Thus, the PD-related DTCs specific to the duration of the late swing phase of gait and gait stability during weight transfer are consistent with the effects of basal ganglia degeneration on SMA connectivity and postural adjustments in people with PD (24, 60–62). While we lack data on cortical activation to make firm conclusions about the nature of the DTC-related deficits we observed, we speculate that the PD-related DTCs during the late swing phase and weight transfer (λ_HC_) may be indicative of greater cortical involvement for locomotion in PD, due to reduced automaticity (7, 8). In people with PD, reduced automaticity and increased cortical control over action has been put forward to explain DT costs during gait (23). Our results suggest that DT interference, possibly due to increased reliance on cortical control in PD, may be more likely to occur at specific phases of gait that normally require cortical activity for stabilization, such as during late swing and weight transfer, as there may be less cognitive resources available for concurrent tasks at these phases.

We compared several spatiotemporal measures and measures of stability, but only phase-dependent LDS at weight transfer (λ_HC_), and the time spent in late swing immediately prior to weight transfer, demonstrated PD-related DTCs. This result agrees with Rochester et al. (23), who similarly found differences in postural stability measures of step width, but failed to find PD-related differences in step length, step time, or step velocity. Furthermore, these results suggest that DT assessments may ignore temporal variation in the demands of the locomotor task. While several models of DT interference exist (e.g., bottleneck, resource limiting, and cross talk) (21), an implicit assumption across all models is that the two competing tasks occur simultaneously and uniformly. While studies have investigated how different cognitive tasks with variations in attentional focus over time influence DT costs during gait (63–65), few studies have examined the temporal variation of the demands of the locomotor task. Nonetheless, the idea that attentional demands vary across a gait cycle was suggested early on by Lajoie et al. (66), who found verbal reaction time was slower during single support compared to double support phases of the gait cycle. However, while Lajoie et al. (66), found reaction time varied by gait cycle in healthy young adults, they did not find DT differences in gait variables when assessing entire strides such as cadence, stride length, stride time, and gait speed. The general lack of consideration for intra-stride changes related to the locomotor task may help explain why DT assessments of gait have little added value over ST assessments when predicting future falls (67, 68). Supporting this notion, we found PD-related, DT interference on LDS only at a specific phase of gait, weight transfer. However, it is possible that severe PD-related DTCs, even if occurring only around weight transfer, could manifest in spatiotemporal measures of whole strides. Other studies have found PD-related DTCs in a variety of spatiotemporal measures, but there is variability about the magnitude of the effect and which spatiotemporal measures are affected (25). A phase-specific DTC in people with PD may explain some of this variability, where the PD-related DTC is blurred across the entire stride and only large magnitude DTCs are measurable. Combined, these results suggest that assessments should examine specific phases of gait, and that targeted interventions should specifically focus on improving the automaticity of foot placement and weight transfer during gait.

Few, if any, studies have compared phase-dependent LDS between people with PD and healthy controls. Yet, our results agree with previous studies that found phase-dependent LDS differences between young and older adults (33) and elderly fallers and non-fallers (34) specific to the weight transfer phase. Notably, older adults had larger λ_HC_, indicative of less stable dynamics, than young adults during steady-state treadmill walking (33). In a later analysis of data obtained during uncontrolled walking, Ihlen and colleagues (34, 35) found elderly with a history of falls had lower λ_HC_ values than non-fallers during daily living activities, indicating more stable dynamics. This disparity was attributed to fallers engaging in less complex tasks at home. A separate analysis found λ_HC_ increased with increasing gait speed (32), suggesting gait speed may have played a role in the lower λ_HC_ values in fallers compared to non-fallers. In our study, the control subjects decreased λ_HC_ and slowed down when walking with a cognitive task, while individuals with PD slowed down but did not proportionally change λ_HC_. Therefore, after adjusting for changes in gait speed, our results can be interpreted similarly to the previous studies on steady-state gait and aligns with the larger body of literature on LDS, where larger λ values indicate less stability (47). Accordingly, cognitive tasks during gait induced less stable dynamics during weight transfer in people with PD compared with similar-aged, elderly control subjects.

While this study benefited from a large sample size, several limitations should be considered when generalizing the results. First, the analysis of LDS and phase-dependent LDS was performed on a small number of consecutive strides. The small number of stride may have increased the within-subject variability across bouts which was partially mitigated by obtaining many bouts of gait (44). In preliminary analyses, we excluded 26 subjects with PD and 16 control subjects who had less than four bouts—21% of the current sample—and we found identical results as presented here, suggesting that the current results are robust; the results do not appear to be driven by subjects with a small number of bouts. However, the present conclusions could be strengthened in future analyses considering a greater number of, and longer, bouts of consecutive strides.

Second, all subjects performed the same cognitive task, which introduced two confounding variables: between-subject differences in cognition and temporal variations in cognitive load as mentioned earlier. The PD group had significantly fewer correct responses across both seated and walking tasks, despite similar total responses, suggesting that our results may be associated with cognitive differences between groups. Yet, the DTC of λ_HC_ was not associated with the MoCA or the SCOPA-COG within either group, suggesting that cognitive differences alone do not explain our results. Furthermore, the lack of a significant group*task interaction for any of the cognitive outcomes suggests that the PD group did not prioritize the cognitive and motor tasks differently than controls. It is possible the fixed order of the conditions may have introduced an order effect. However, the order was consistent across groups and the primary inferences were drawn from the group*task interaction. Similarly, the difference in duration between the walking conditions (2 min ST versus 1 min DT) led to fewer strides and bouts within the DT condition. The shorter DT duration was selected to accommodate people with PD who had difficulty completing 2-min of continuous DT walking. It is possible the different durations influenced the main effect of task, but it is unlikely the main inferences drawn from the group*task interaction were affected.

Finally, it is unclear how freezing of gait influenced our results. While bouts of gait that included a freezing episode were excluded from any analysis, it is unclear whether people PD with and without freezing of gait differed in bouts without a freezing episode. The relatively small number of people with PD who exhibited freezing of gait in our sample prevented a sub-analysis examining this question. However, future studies may investigate whether freezing of gait is similarly associated with phase-dependent DT costs.

Overall, these results challenge our understanding of DT costs while walking, particularly in people with PD. With growing evidence that cortical control occurs during specific phases of gait, it is necessary to re-evaluate traditional concepts of DT interference that may neglect the phasic structure of control during locomotion. Our results suggest that PD-related DT interference occurs only immediately before and during postural adjustments at weight transfer. Interventions, particularly those utilizing DT and multi-task training paradigms, may benefit from focusing on postural adjustments during gait, and future research should directly examine this question using mobile neuroimaging modalities time-locked to phases of the gait cycle.

## Ethics Statement

This study was carried out in accordance with the recommendations of the Oregon Health & Science University (OHSU) and Veterans Affairs Portland Health Care System (VAPORHCS) joint institutional review board (IRB) with written informed consent from all subjects. All subjects gave written informed consent in accordance with the Declaration of Helsinki. The protocol was approved by the OHSU (#4131) and the OHSU/VAPORHCS joint IRB (#8979).

## Author Contributions

PF, MM, and FH conceptualized the question and hypothesis. FH, JN, and MM designed the study from which the data originates. PF, MM, and CC contributed to data collection and analysis. PF, MM, CC, JN, and FH contributed to the interpretation, writing and editing of the manuscript. PF wrote the first draft.

## Conflict of Interest Statement

FBH has a significant financial interest in APDM, a company that may have a commercial interest in the results of this research and technology. This potential conflict has been reviewed and managed by OHSU. All other authors declare that the research was conducted in the absence of any commercial or financial relationships that could be construed as a potential conflict of interest.
